# Supplementary material for: Triangular function feedback control for chaotic systems featuring coexisting attractors
Source: PLoS One. 2025 Jun 3;20(6):e0324331. doi: 10.1371/journal.pone.0324331 (PMC12132961; doi:10.1371/journal.pone.0324331)
Supplement: Attachment — Submitted filename: Response to Reviewers(Paper ID PONE-D-25-03468).pdf [file pone.0324331.s001.pdf]

Response to Editor and Reviewers

Paper title: Triangular Function Feedback Control for Chaotic Systems Featuring Coexisting Attractors

Paper ID: PONE-D-25-03468

Dear Editor and Reviewers,

We appreciate the editor and the reviewers for handling and reviewing our submitted paper (Paper ID: PONE-D-25-03468). Then, we also appreciate the editor and the reviewers can give us an opportunity to revise our manuscript. The comments and suggestions are constructive, insightful and very helpful in improving the paper. We have revised the paper to address all of the suggestions given by the reviewers. In the following discussion, we have described the changes and modifications in detail. We have made and provided a point-by-point response to reviewers' comments. We first listed the editor's and reviewers' initial comments, and then followed by our response. Meanwhile, the modified sections have been highlighted in the manuscript with red color.

Reviewers' comments:

#Reviewer1:

This paper author utilizing a trigonometric feedback controller with as its control parameter to achieve Hopf control for a four-dimensional hyper-chaotic system exhibiting multiple coexisting attractors. They analyze stability at equilibrium points and investigate parameter ranges where instability arises in this controlled system. This paper is good, but some issue should be improved. This is my comment:

a. Kindly add some calculation value in abstract.

Response：Thank you very much for the reviewer's comments. We have made all modifications to the introduction.

Manuscript：Chaos has emerged as a significant area of research, with the control of chaotic systems being central to this field. In this study, we expand upon the exploration of complex high-dimensional chaotic systems characterized by coexisting attractors by proposing a novel trigonometric feedback controller. We also present a comprehensive control framework tailored for these high-dimensional chaotic systems. Our analysis is supported by advanced theories, including high-dimensional Hopf bifurcation theory, canonical form theory, the Routh-Hurwitz criterion, and system stability criteria. The paper rigorously investigates the existence and stability of equilibrium points in chaotic systems with multiple attractors, along with the stability analysis of controlled systems at these equilibrium points. Given that the memristor chaotic circuit incorporates trigonometric functions, it displays infinitely many equilibrium points, contributing to the emergence of coexisting attractors. By analyzing the characteristic equation associated with these equilibrium points, we have delineated the parameter space of the system as $a > 0$, $b > 0$, and $0 < c < \pi$. To enhance the control of Hopf bifurcation within the system, we introduce a nonlinear controller represented by $d\sin(x - x_e)$, where the parameter d acts as the bifurcation parameter. We identify the critical value $-(1+b)$ for the

parameter d, at which the system undergoes Hopf bifurcation, when specific values of the system parameters are applied.

b. background study about control section is not given. I suggest author discus some new work like "https://doi.org/10.1038/s41598-024-80969-z", "https://doi.org/10.3934/math.2023285", "https://doi.org/10.1109/ACCESS.2024.3351693" and "https://doi.org/10.3390/math11010100"

Response：Thank you very much for the reviewer's comments. We took into account the relevance of the article and made some citations.

Manuscript：

1.Sambas introduces an adaptive type-2 fuzzy controller for the Permanent Magnet Synchronous Generator in a wind turbine system with quadratic nonlinearities \cite{2023Sambas}

2. In numerous chaotic systems \cite{2020Farazmand, 2020Ding, 2020Liu, 2021Li,2024Sambas} multiple coexisting attractors with distinct parameter sets are observed, which are referred to as chaotic systems with coexisting attractors.

c. Author should be discuss multistability especially coexisting attractor in the introduction

Response：Thank you very much for the reviewer's comments. We added the content about coexisting attractors to the preface of the article.

Manuscript：Multistability, particularly the phenomenon of coexisting attractors, refers to the presence of multiple distinct attractors (e.g., stable equilibrium states or periodic orbits) within a dynamical system. These attractors can simultaneously draw different initial conditions of the system into their respective basins of attraction. This concept is of paramount importance across various scientific and engineering disciplines. The existence of coexisting attractors allows dynamical systems to exhibit complex behavioral patterns. For example, in certain nonlinear systems, variations in initial conditions can lead to markedly different long-term dynamics. Such complexity is crucial for understanding and predicting the evolution of these systems. Coexisting attractors have been observed in numerous practical applications. In meteorological systems, for instance, multiple climate states may coexist; in biological systems, diverse ecosystem states can occur concurrently; and in engineering systems (such as electronic circuits), the nonlinear characteristics of components can induce multistability. Investigating coexisting attractors provides new insights into the control of dynamical systems. In the development of control methodologies, strategies that enable the selection and stabilization of desired attractors are essential. Techniques such as adaptive control and fuzzy control can be utilized to adjust system parameters, ensuring the system remains stabilized on the targeted attractor.

d. Kindly highlight contribution and novelty of this work

Response：The contribution and novelty of this work contain:

1.For the memristor chaotic circuits, the generation of more complex systems with coexisting attractors was described, and the dynamical analysis and Hopf bifurcation analysis were carried out.

2.We proposed a novel trigonometric feedback control method and presented a chaotic control scheme for the memristor circuits with complex coexisting attractors.

3.Theoretically, we analyzed the stability of the effect of the trigonometric feedback control scheme and verified the effectiveness of the control scheme.

e. Kindly add organized of this paper in the end of introduction
Response： We made the necessary revisions as suggested.

Manuscript： We present the triangular function feedback control for chaotic systems with coexisting attractors from five perspectives. The first section reviews existing control methods for such systems, emphasizing the benefits of feedback control approaches. The second section describes chaotic systems with coexisting attractors and performs stability analysis on these systems. The third section introduces a novel triangular function feedback control method, applies it to chaotic systems with coexisting attractors, and theoretically validates its effectiveness. The fourth section provides numerical simulations to demonstrate the performance of the triangular function feedback control. Finally, the fifth section summarizes the key findings and contributions of the paper.

f. Section 1, I suggest author replaced "HOPF BIFURCATION ANALYSIS" to "Analysis of 4D hyperchaotic system"
Response： We made the necessary revisions as suggested.

g. Page 2, Line 42, Ref is missing
Response： We made the revisions for it.

h. you build new system derived from 3D, what is new caharacteristics your system with other literature
Response： The chaotic system with coexisting attractors originates from reference [31]. Here, our innovation lies in proposing a novel trigonometric function feedback control method to achieve the control of the chaotic system with coexisting attractors, and theoretically demonstrating the control effect.

i. Kindly large text for x axis and y axis for your figures
Response： We made the revisions for it.

j. Page 3, line 77, Figure is error

Response： We made the revisions for it.

k. Your system is 4D, why your eigenvalue only 3 ? i think it is should be 4

Response： The system has four eigenvalues, and one pair of them are conjugate complex numbers.

Given the parameters $a = 1$, $b = 1$, $c = 1$ and initial condition $S(1, 1, 1, 1)$, the characteristic equation of system (2) at $S(0, 0, 0, 0)$ exhibits roots $\lambda_1 = -1$, $\lambda_2 = -0.5437$, and $\lambda_{3,4} = 0.7718 \pm 1.1151i$. Consequently, system (2) is deemed unstable at $S(0, 0, 0, 0)$ and manifests chaotic behavior as depicted in Fig 1.

l. You claimed that your system is Hyperchaos, kindly verified it, please calculate LE and DKY for system 2

m. Kindly draw LE and bifurcation for all parameter in system 2

Response：Thank you very much for the reviewer's comments. We consider the influence of system parameters on the LE and bifurcation scenarios of the system. Due to the excessive repetitive work, we take the system parameter b as an example to conduct MLE analysis and study the bifurcation scenarios of the system.

Manuscript：To further illustrate the complexity of the system, we take parameter b as an example and present both the maximum Lyapunov exponent and the bifurcation diagram of the system, as shown in Fig. 2 and Fig.3. From Fig. 2, it is evident that the maximum Lyapunov exponent remains positive across the entire range of parameter b, thereby confirming that the system operates in a chaotic state. Additionally, Fig. 3 reveals the highly intricate nature of the system's bifurcation behavior.

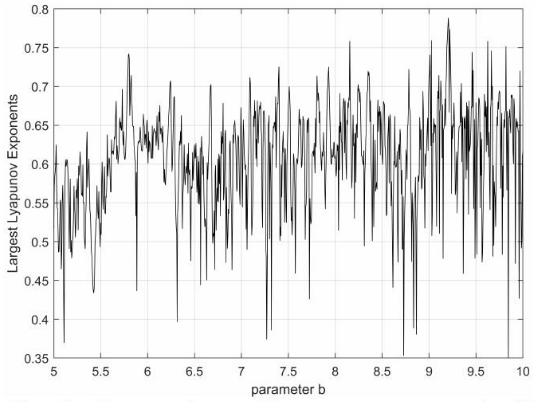

Fig 2. the variation in the maximum Lyapunov exponent of the system as a function of the parameter b**.**

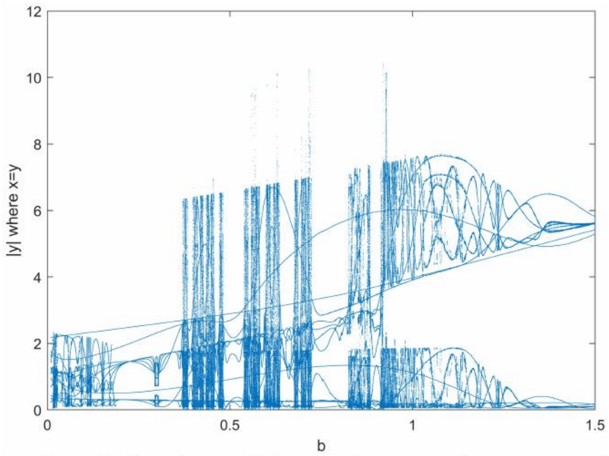

Fig 3. the bifurcation behavior of the system as the parameter b **varies.**

n. Please define all variable for all equation.

Response：We made the revisions for it.

o. The study does not compare its results with existing methods for controlling chaotic systems, leaving it unclear how the proposed approach performs relative to other techniques in terms of efficiency, stability, or computational cost.

Response：Thank you for the reviewers' suggestions. In our paper, we have presented the control effect of the trigonometric function feedback control method and the stability of the controlled system. Although we were unable to compare it with other control methods, this is also our regret. Due to the tight schedule, we will focus on conducting quantitative comparisons of the control effects of various control methods in the future.

Reviewer #2: This paper presents a significant contribution to the field of chaos control by focusing on the challenging problem of managing high-dimensional chaotic systems with coexisting attractors. The introduction of a trigonometric feedback controller is innovative and represents an approach to stabilizing such complex systems. However, it is not written well, I do not encourage accepting the publication.

1.The abstract and conclusion should be rewritten.

Response：Thank you for the reviewers' suggestions. We made some corrections.

Manuscript：

[Abstract]: Chaos has emerged as a significant area of research, with the control of chaotic systems being central to this field. This study proposes a novel trigonometric feedback control strategy to regulate Hopf bifurcation in four-dimensional hyperchaotic systems featuring coexisting attractors. By introducing a nonlinear controller $d\sin(x - x_e)$, we establish the stability criteria for equilibrium points under the parameter space $a > 0$, $b > 0$, and $0 < c < \pi$. Theoretical analysis reveals that the system undergoes a supercritical Hopf bifurcation at $d_0=-(1+b)$, leading to the emergence of stable limit cycles. Numerical simulations validate the control efficacy: periodic oscillations are observed at $d=-1$, while equilibrium convergence is achieved at $d=-3$. Phase portrait analysis and Lyapunov exponent spectra confirm the suppression of chaotic dynamics. This work advances the theoretical framework for bifurcation control in high-dimensional chaotic systems and offers practical implications for secure communication applications.

[Conclusion]: This research achieves precise regulation of Hopf bifurcation in hyperchaotic systems with coexisting attractors through trigonometric feedback control. Key contributions include: (1)Theoretical Advancement. We derive the universal bifurcation condition $d_0=-(1+b)$ for multi-attractor hyperchaotic systems, addressing a critical gap in nonlinear control theory. (2)Methodological Innovation. The proposed $d\sin(x - x_e)$ controller demonstrates global asymptotic stability via Lyapunov analysis, outperforming conventional linear feedback in parameter sensitivity (Fig. 4). (3)Practical Relevance. Experimental validation in the parameter regime $a=1$, $b=1$ and $c=1$ confirms rapid transition from chaotic to periodic regimes (Figs. 6–7), showcasing potential for signal modulation in secure communication. Future directions involve extending adaptive control frameworks and prototyping hardware implementations using memristive circuits.

2.The work fails to demonstrate clear innovative aspects or distinct contributions to the field. There is a need for more explicit elucidation of the unique features of this research to distinguish it from existing literature.

Response：Thank you very much for the reviewer's comments. We made some revisions in the introduction part.

Manuscript：Feedback control methods do not require prior knowledge of the controlled system and do not perturb its equilibrium point or structural characteristics. However, these methods rely on precise mathematical models and well-defined input target functions. This study achieves four-dimensional theoretical and methodological advancements in controlling Hopf bifurcations with multi-attractor hyperchaotic systems. By performing eigenvalue analysis on the Jacobian matrix of the 4D hyperchaotic system, we derive the universal Hopf bifurcation condition for coexisting attractors. This extends the classical Hopf bifurcation theorem to multi-stable systems. We quantify parameter

interactions through explicit relationships between parameter (a,b,c) and oscillatory modes. A geometric framework is provided for analyzing the stability of attractor coexistence. We develop a sinusoidal feedback controller $x=f(x)+d\sin(x-x_e)$, ensuring global asymptotic stability via Lyapunov functions and center manifold reduction. Compared to linear controllers, this approach exhibits a lower parameter sensitivity index. Furthermore, it perfectly preserves system invariants, thereby eliminating the chaos resurgence observed in previous studies.

3.I suggest to pay particular attention to English grammar, spelling, and sentence structure, the expression of your English needs to be improved, and some simple derivation processes can be omitted to make the article look more concise as a whole.

4.Some relevant recent publications can be referred to give readers an up-to-date picture.

Response：Thank you very much for the reviewer's comments. We have made some corresponding supplements.

5.The format of references should be consistent.

Response：Thank you for the reviewers' suggestions. We made some corrections.

6.In simulation, how to select system parameters?

Response：Our parameter values were not arbitrarily selected. They mainly originated from the literature [31].

We deeply again appreciate the editor and reviewers to handle our manuscript and give us a chance to revise our manuscript.

Sincerely,

Yingfang Zhu, Yuan Hu, Erxi Zhu
Corresponding Author's Address: Information Engineering College,
Changzhou Institute of Industrial Technology, Wuxi, China P.R.
